# Spatiotemporal Redox Regulation of Cell-Cycle Progression: Dynamic Functions of Cellular Redox Regulatory Proteins

**DOI:** 10.3390/cells15191814

**Published:** 2026-10-04

**Authors:** Raghavendra S Patwardhan, Shruti Morjaria, Gargi Nandi, Syed Hasan, Santosh K. Sandur

**Affiliations:** 1 Radiation Biology & Health Sciences Division, Bhabha Atomic Research Centre, Trombay, Mumbai 400085, India; 2 Homi Bhabha National Institute, Training School Complex, Anushaktinagar, Mumbai 400094, India; 3 Radiobiology & Omics Lab, Radiological Research Unit, Bhabha Atomic Research Centre Facility, Kharghar, Navi Mumbai 410210, India; 4 Advanced Centre for Treatment, Research & Education in Cancer (ACTREC-TMC), Kharghar, Navi Mumbai 410210, India

**Keywords:** redox, cell cycle, ROS, GSH

## Abstract

**Highlights:**

**What are the main findings?**

- Redox regulation is dynamically coordinated with cell-cycle progression through phase-specific changes in H_2_O_2_ generation, sensing, buffering, and resolution.
- MnSOD, peroxiredoxins, Nrf2/HO-1/NQO1, glutathione, glutaredoxins, GPXs, and the thioredoxin system perform complementary functions that link redox homeostasis with proliferation, DNA replication, checkpoint control, and mitosis.

**What are the implications of the main findings?**

- Cell-cycle progression depends on maintaining a defined redox signaling window that permits physiological H_2_O_2_ signaling while limiting oxidative damage.
- Disruption of this coordinated redox network may contribute to genomic instability, aberrant proliferation, and cancer-cell survival, highlighting redox vulnerabilities as potential therapeutic targets.

**Abstract:**

Aerobic life revolves around the enigmatic role of oxygen, which sustains the life cycle by supporting energy production while simultaneously threatening its existence through the generation of reactive oxygen species (ROS). Intriguingly, ROS are not merely by-products of energy generation; cells actively invest in their production through dedicated pro-oxidant systems. A long-standing perception in the scientific community has been that cellular antioxidant systems primarily evolved as terminators of ROS to protect against oxidative damage. It appears paradoxical, however, that the complex, multilayered antioxidant defense built by cells may actually relay, propagate, and buffer ROS in a spatiotemporal manner rather than simply scavenge them. Perpetuation of life essentially depends upon the cell cycle, one of the most orderly and tightly regulated cellular events. While several elegant reviews have highlighted that cell-cycle regulatory proteins are subject to redox modifications and that changes in cellular redox state influence cell-cycle fate, the converse relationship has received relatively less attention. Proteins regulating cellular antioxidant defense themselves undergo cell-cycle phase-specific changes in their abundance, activity, and localization. There therefore appears to be a coordinated and integrated redox network operating during cell-cycle progression. Here, we examine this relationship from a protein-centric perspective, focusing on MnSOD/H_2_O_2_, peroxiredoxins, Nrf2, HO-1, NQO1, glutathione, glutathione peroxidases, glutaredoxins, and the thioredoxin/thioredoxin reductase system. Although these systems have often been studied independently, their functions overlap and change as cells move through G1, S, G2, and mitosis. We propose that cell-cycle progression should be viewed as a dynamically regulated redox process in which spatiotemporally organized oxidative signals provide an additional regulatory layer operating alongside the classical cell-cycle machinery.

## 1. Introduction

Over the past two decades, it has become increasingly apparent that the classical cell-cycle machinery does not operate independently of cellular redox metabolism [1,2,3,4]. Interestingly, it appears that cells integrate information about intracellular redox balance to coordinate DNA synthesis, chromosome segregation, and checkpoint signaling. This provides a fresh perspective to our understanding of reactive oxygen species (ROS) as highly regulated signaling molecules that participate in cell-cycle regulatory network.

Among the various ROS, hydrogen peroxide (H_2_O_2_) has emerged as a key redox messenger because of its long half-life, diffusibility, and ability to reversibly oxidize reactive cysteine residues [5]. It is notwithstanding that oxidation of selected cysteine residues modulates the activity of phosphatases, kinases, transcription factors, and DNA repair proteins, thereby coupling metabolic activity with cell-cycle progression [6,7,8,9,10,11]. These discoveries emphasize that oxidation–reduction reactions constitute an additional regulatory layer superimposed upon conventional signaling networks.

Multiple studies have shown that ROS concentrations oscillate as cells transition between quiescence, DNA replication, and mitosis [12,13,14,15]. Careful examination of the literature also reveals that antioxidant systems also undergo coordinated changes in abundance, catalytic activity, intracellular localization, and protein–protein interactions [1,16,17,18]. In the context of ROS oscillations, it is important to note that these dynamic changes in antioxidant systems together determine when, where, and for how long redox-dependent signaling can occur [19,20,21,22,23]. If viewed within a single framework, it appears reasonable and biologically plausible that, in fact, antioxidant systems are critical mediators of redox signaling and do not simply perform indiscriminate scavenging of ROS [24].

Several comprehensive reviews have elegantly discussed how ROS signaling is closely intertwined with cell cycle and that cell cycle is a redox cycle [2,3,4,17,18,25]. Sies et al. and Burhans et al. have elegantly reviewed how cellular redox state changes during each phase of the cell cycle [3,25]. Further, multiple studies have revealed an intricate relationship between cellular antioxidant proteins and cell-cycle progression [2,26,27,28]. However, there has been no concerted effort to understand the coordinated behavior of redox regulatory proteins themselves throughout successive phases of the mammalian cell cycle.

In this review, we propose that cell-cycle progression is governed by an integrated and highly dynamic redox regulatory network. Rather than focusing exclusively on ROS production or oxidative stress, we examine how major redox regulators—including Nrf2, MnSOD, HO-1, NQO1, peroxiredoxins, glutathione, glutathione peroxidases, glutaredoxins, and the thioredoxin/thioredoxin reductase system—undergo coordinated changes in expression, localization, activity, and function as cells progress through the cell cycle (Table 1). This is a hitherto unique effort to prepare a collage of redox regulatory proteins and integrate these observations into a systems-level framework that places redox regulation alongside canonical cell-cycle signaling.

## 2. Redox Signaling: An Additional Regulatory Layer of Cell-Cycle Progression

Cell division requires continuous metabolism, and ROS are inevitably generated via mitochondrial respiration, NADPH oxidases, and several oxidoreductases. Instead of removing these oxidants from the intracellular environment, proliferating cells actively maintain an intracellular ROS level that allows signaling but does not reach the level that causes irreversible oxidative damage [51,52]. Such a fine balance indicates that ROS are not mere metabolic byproducts but essential players in the regulatory network that controls cell-cycle progression. Indeed, ROS are embedded in a highly sophisticated signaling network in which H_2_O_2_ appears to be a carrier of intracellular redox information [53].

From the classical oxidative stress paradigm to modern redox signaling, the transition did not occur merely because new ROS had been identified. It was due to the realization that H_2_O_2_ reversibly modifies specific cysteine residues in strategically important positions in phosphatases, kinases, transcription factors, and metabolic enzymes, thus exerting regulatory effects that very much resemble those of reversible phosphorylation [53,54,55,56]. In our view, this is one of the elegant examples of how evolution may have retooled a potentially damaging molecule into a versatile currency for intracellular signaling.

One of the most pertinent conceptual advances in recent years has been the idea that H_2_O_2_ signaling depends not on an absolute peroxide level but on its spatial and temporal organization [55]. Several enzymatic systems, including mitochondrial respiration, NADPH oxidases, xanthine oxidase, and several flavin-containing oxidoreductases, continuously generate H_2_O_2_ in the cell [57]. However, this peroxide almost never accumulates homogeneously within the cytoplasm. Instead, localized H_2_O_2_ microdomains are formed in specific intracellular compartments where transient oxidation of selected proteins may occur without disturbing the overall redox homeostasis [58,59]. Such compartmentalization provides the specificity that allows peroxide to regulate proliferation without causing massive oxidative damage.

Such localized peroxide gradients become relevant during cell-cycle progression. For example, entry into the cell cycle requires growth-factor signaling, which depends on the transient oxidation of protein tyrosine phosphatases. During the early G1 phase, growth factor stimulation activates membrane-associated NADPH oxidases, leading to the localized generation of H_2_O_2_, which transiently inhibits phosphatases such as PTP1B [60]. Such transient phosphatase inhibition prolongs receptor tyrosine kinase signaling to activate the MAPK and PI3K-AKT pathways, induce Cyclin D (CDK), and progress toward the restriction point.

Instead of eradicating peroxide, proliferating cells allow controlled oscillations in H_2_O_2_ to maintain CDK activation, replication-associated signaling, and metabolic adaptation, while antioxidant systems prevent these signals from surpassing the threshold that would initiate checkpoint responses.

Intracellular H_2_O_2_ levels increase as cells progress toward G2/M, where localized redox signaling contributes to mitotic progression, including centrosomal signaling and the regulation of processes required for accurate spindle function and chromosome segregation [27,39]. Peroxide-mediated local oxidation of phosphatases, such as PP2A and Cdc14B, stabilizes mitotic regulators, including Cyclin B1 and CDK1, to facilitate orderly entry into mitosis. Following mitotic progression and cytokinesis, reactivation of antioxidant systems helps restore intracellular peroxide homeostasis, thereby terminating phase-specific redox signaling and re-establishing the basal redox state in daughter cells [39]. Based on the collective evidence reviewed here, we propose a model in which coordinated changes in ROS generation, peroxide sensing, thiol buffering, and reductive capacity contribute to phase-specific regulation of cell-cycle progression. Although individual components of this network have been experimentally characterized, whether these pathways operate as a fully integrated spatiotemporal circuit during the complete mammalian cell cycle remains to be directly demonstrated. The phase-specific distribution and functional associations of these redox regulators across the cell cycle are summarized in Table 2.

The most considerable misunderstanding in H_2_O_2_ biology is that its biological effects depend only on its level. On the other hand, the literature supports a much more sophisticated view: the effects of peroxide signaling depend on the concentration, time, intracellular localization, and antioxidant status of the microenvironment where it occurs. In this context, moderate and transient increases in H_2_O_2_ promote proliferation; on the other hand, sustained or high peroxide activates ATM- and ATR-dependent DNA damage responses, stabilizes p53 and p21, inhibits cyclin–CDK activity, and induces senescence or apoptosis. These contradictory observations have sometimes caused confusion in the literature. However, they become entirely consistent when peroxide is viewed as a signaling molecule with physiological limits rather than a general cytotoxic oxidant.

Another significant insight from recent research is that peroxide signaling cannot be conceptualized separately from the proteins that regulate it. In many ways, H_2_O_2_ is a signaling intermediate whose biological significance depends on the molecular context in which it is generated. MnSOD continuously supplies mitochondrial peroxide, peroxiredoxins define where peroxide signaling is allowed, glutathione and glutathione peroxidases establish buffering capacity, glutaredoxins regulate reversible thiol modifications, and the thioredoxin system returns oxidized proteins to a functional state. Thus, H_2_O_2_ biological activity reflects the whole redox regulatory network’s integrated behavior.

## 3. Dynamic Redox Regulatory Proteins During Cell-Cycle Progression

### 3.1. Manganese Superoxide Dismutase (MnSOD) as a Regulator of Cell Cycle

Among all other players in the cellular redox regulatory network, manganese superoxide dismutase (MnSOD; SOD2) occupies a very privileged position since it interpenetrates mitochondrial metabolism and intracellular signaling [16,61]. Every proliferating cell must upregulate oxidative phosphorylation to provide the energetic and biosynthetic precursors for DNA replication and cell division. Therefore, increased respiratory flux invariably produces increased superoxide anion (O_2_•^−^) levels at the electron transport chain complexes I and III. For many decades, that superoxide was viewed mainly as an accidental metabolite and, therefore, toxic to mitochondrial membrane integrity and genome stability. That view has since reversed. MnSOD’s expressed patterns have changed dramatically: once viewed as an antioxidant enzyme, there is increasing acceptance today that MnSOD translates mitochondrial metabolism into meaningful redox signaling [16].

This general shift in thinking did not develop overnight. Indeed, initial work by Larry Oberley and co-workers brought the tumor-suppressive activity of MnSOD to light when they showed that MnSOD re-expression suppressed the proliferation of transformed cells [62]. On these data alone, a mode of action of MnSOD based on protective elimination of harmful reactive oxygen species (ROS) was built through subsequent studies by Daret St Clair, Frederick Domann, and others. The role of MnSOD in transmuting superoxide into hydrogen peroxide was later established. Thus, MnSOD does not detoxify one form of reactive oxygen species (ROS) but transmutes one form of redox information into another [33]. Indeed, increased MnSOD activity would favor a shift in mitochondrial redox chemistry toward hydrogen peroxide (H_2_O_2_)-dependent signaling pathways. In comparison, decreased MnSOD activity would allow the superoxide level to continue unabated.

Changes in redox status driven by MnSOD indirectly change the signals that determine cell-cycle progression through diverse signaling pathways. Increased MnSOD activity is associated with decreased Cyclin D1, Cyclin E, CDK2, Cyclin B1, and proliferating cell nuclear antigen (PCNA) [63,64]. Conversely, decreased MnSOD activity accelerates the cell cycle through Cyclin D1 and Cyclin B1 expression, promoting proliferation and G2/M accumulation [65]. Indeed, MnSOD is again embedded in a more comprehensive regulatory loop: NF-κB upregulates MnSOD when mitochondrial protection is required, but during “active” growth, proliferative transcription factors c-Myc and E2F1 repress MnSOD expression and allow for a transient rise in mitochondrial reactive oxygen species (ROS) that aids proliferation [32,66]. Such reciprocal regulation is consistent with MnSOD being embedded in an adaptive feedback loop rather than operating as a constitutively active antioxidant. The clearest evidence of this comes during the quiescence–proliferation transition. While G0 and early G1 cells maintain relatively high Mn-SOD activity, this forms a basal level of mitochondrial superoxide compatible with a redox state for reversible growth arrest [16,65,67]. Upon experimentally lowering MnSOD, this balance is lost with increased superoxide and associated increased expression of Cyclin D1, impairing the entry of cells into quiescence [65]. Such changes are reversible after restoration of MnSOD, which favors re-entry into a proliferative state. The reported cell cycle-associated targets of MnSOD and other redox regulators are summarized in Table 3.

### 3.2. Context-Dependent Functions of Mn-SOD

On the other hand, it seems hardly conclusive: How can one reconcile the doctrine of MnSOD-mediated antiproliferative effects with the presence of considerable MnSOD activity in proliferative cells? How does one reconcile the doctrine that decreased MnSOD promotes cell-cycle progression with the observed phenotype of impaired proliferation upon total MnSOD loss? We consider the progressively different findings to converge on the hormetic redox paradigm involving MnSOD. Physiological proliferation during the cell cycle does not demand peak antioxidant defense or, conversely, maximal reactive oxygen species (ROS) production. Instead, it depends on a precise, well-defined signaling window of mitochondrial reactive oxygen species (ROS). An over-abundance of MnSOD would suppress superoxide-dependent mitogenic signaling; less MnSOD would allow unchecked oxidative stress, triggering a checkpoint response and damaging mitochondria. Therefore, the association is not linear but biphasic, with a peak in proliferation occurring only with tight regulation of mitochondrial reactive oxygen species (ROS). This is evident moving forward at G1/S. Passing the restriction point is probably the most metabolically demanding stage throughout the entire cell cycle; hence, metabolic reprogramming needs to be coordinated with cyclin–CDK activation. Most evidence claims that mitochondrial reactive oxygen species (ROS) are transiently needed during this time. Increased MnSOD activity dampened the superoxide-dependent signal that inhibits Cyclin E-CDK2 activity, suppresses proliferating cell nuclear antigen (PCNA) expression, and delays DNA synthesis. On the other hand, reduced MnSOD levels sustain superoxide accumulation without simultaneously triggering oxidative damage, thus reaching a critical threshold at which proliferative signaling overcomes the fidelity-associated damage signals. Hence, the step toward DNA replication is a more dynamic balance concerning MnSOD than a state of heightened antioxidant activity. Metabolic upregulation related to nucleotide biosynthesis, chromatin remodeling, and DNA replication in cells entering the S phase will vastly increase mitochondrial metabolism. Naturally, this implies an increase in oxidative phosphorylation and, therefore, an increase in electron leakage in the respiratory chain with a concomitant increase in reactive oxygen species (ROS) production. Evidence, however, shows that, in this period, MnSOD activity, or at least its effective activity, is at a minimum, allowing the mitochondria to mount the peroxide-dependent signal to enable DNA synthesis while the cell places reliance downstream on glutathione, peroxiredoxins, and the thioredoxin systems to mitigate the increased oxidative damage [16,33,63,68]. This signals that the mitochondria are unable to effectively time these metabolic transitions in MnSOD-deficient cells, thus implicating redox control as an essential factor in bioenergetics requirements during genome duplication. Correspondingly, reduced MnSOD levels extend into later mitotic phases by accumulating cells at the G2/M boundary with increased Cyclin B1, wherein excessive mitochondrial superoxide disrupts orderly progression into mitosis. This block can be released by replacing MnSOD [69,70]. Thus, proliferating cells are endowed with intracellular glutathione-raising instead of MnSOD during mitosis, illustrating again how mitochondrial and cytosolic antioxidant activities are not parallel or independent but compensatory, as they follow changing redox demands through the phases of the cell cycle. Therefore, the specific findings actually support the general assumption that no single antioxidant pathway drives the cell cycle alone but rather as an interactive process among several components of the redox landscape. These apparently divergent effects of MnSOD are summarized in Table 4, which highlights the dependence of its biological effects on proliferative state and cellular context. Despite these insights, specific burning issues about MnSOD biology still prevail. MnSOD biology has mostly been studied in immortalized cells/overexpression models; thus, the data might represent adaptation to chronic artificial treatments rather than a physiological state. Whether similar MnSOD oscillations occur in vivo during embryogenesis, tissue regeneration, or immune stimulation remains to be determined, as is the mechanistic basis of the coordination between MnSOD and other redox network participants in sequence through the phases of the cell cycle. Such elicitation will involve live-cell redox imaging, quantitative proteomics, and metabolic flux analysis capturing redox dynamics with improved spatial–temporal resolution.

**Figure 1:**
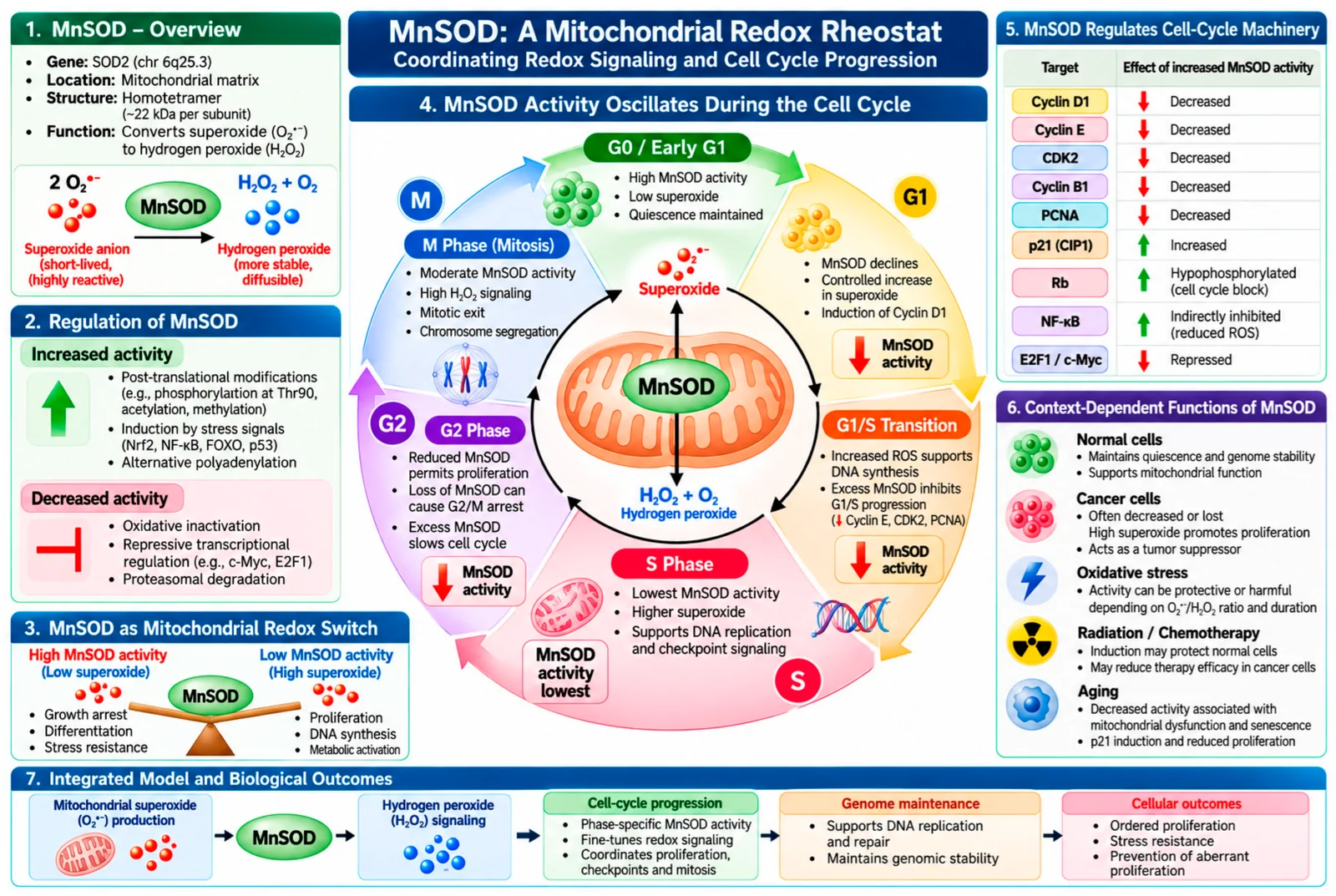
Mn-SOD: A first regulatory node in cell-cycle regulation. (Different colors distinguish cell-cycle phases and functional modules for visual clarity and do not represent quantitative differences. ↑, increased; ↓, decreased.).

Taken together, the accumulated evidence suggests that MnSOD functions beyond its classical role in mitochondrial superoxide detoxification. By controlling the conversion of superoxide to H_2_O_2_, MnSOD influences mitochondrial redox balance and is associated with changes in cell-cycle progression and cellular metabolism. Based on these observations, we propose that MnSOD may represent an upstream regulatory component of the cellular redox network, linking mitochondrial ROS metabolism with proliferative control. We propose that the earliest redox decision governing mammalian cell-cycle progression is made within mitochondria, where MnSOD determines not how much ROS a cell contains, but what kind of redox information is transmitted to the rest of the cell (Figure 1).

### 3.3. Peroxiredoxins (PRXs): The Redox Signal Interpreters That Define Specificity in Cell-Cycle Progression

One of the longstanding questions in redox biology has been how a relatively simple molecule such as hydrogen peroxide can selectively regulate hundreds of signaling pathways without causing indiscriminate oxidative damage. The answer has gradually emerged through the recognition that peroxide signaling is not governed by hydrogen peroxide alone but by an intricate network of peroxide-reactive proteins that determine where, when, and to what extent oxidative signals are transmitted [71]. Among these proteins, the peroxiredoxin family has emerged as one of the most important determinants of signaling specificity [72].

For many years, peroxiredoxins were regarded simply as highly abundant antioxidant enzymes responsible for eliminating intracellular hydrogen peroxide. Seminal work from Sue Goo Rhee, Tobias Dick, and numerous subsequent investigators demonstrated that peroxiredoxins participate directly in peroxide-mediated signaling by undergoing reversible oxidation of their catalytic cysteine residues [73,74]. Prxs undergo reversible phosphorylation, hyperoxidation, glutathionylation, oligomerization, and changes in protein–protein interactions that transiently suppress their peroxidase activity at selected intracellular locations [58,75,76,77,78]. This reversible inactivation allows localized accumulation of H_2_O_2_ precisely where peroxide signaling is required while preventing widespread oxidative damage [79]. Rather than terminating redox signals indiscriminately, it appears that Prxs function during intracellular peroxide signaling, determining the magnitude, duration, and localization of H_2_O_2_-mediated signaling events [76,77].

They function to transfer oxidative equivalents to selected target proteins through reversible thiol–disulfide exchange reactions [77]. Such relay mechanisms enable hydrogen peroxide to modify transcription factors, phosphatases, kinases, and metabolic enzymes with remarkable specificity despite its relatively simple chemical structure [74]. In our view, this represents one of the most elegant solutions evolved by mammalian cells to reconcile the dual requirements of utilizing ROS for signaling while simultaneously preventing oxidative damage.

Entry into the proliferative cycle is accompanied by transient increases in intracellular peroxide that regulate numerous components of the cell-cycle machinery, including protein tyrosine phosphatases, CDK1 (Cdc2), Cyclin B1, Cdc14B, APC/C–Cdh1, FOXM1, JNK/c-Jun, and proliferating cell nuclear antigen (PCNA) [27,40]. However, unrestricted propagation of these oxidative signals would inevitably activate checkpoint pathways and compromise genomic stability. Peroxiredoxins prevent this outcome by rapidly buffering peroxide within defined intracellular microdomains while permitting localized oxidation of carefully selected signaling proteins [71].

During G1 phase, growth factor stimulation generates transient H_2_O_2_ that promotes receptor tyrosine kinase signaling by reversibly oxidizing protein tyrosine phosphatases such as PTP1B [60,80].

During periods of increased peroxide production, catalytic cysteine residues within PRX1 and PRX2 undergo transient hyperoxidation, temporarily inactivating their peroxidase activity [74]. Initially interpreted as oxidative damage, this phenomenon is now recognized as an adaptive signaling mechanism. Local inactivation of Prxs permits transient accumulation of hydrogen peroxide within restricted cellular compartments, thereby facilitating oxidation of downstream signaling proteins before sulfiredoxin-dependent repair restores Prx activity [81]. This “floodgate model” [82] elegantly explains how cells generate discrete peroxide pulses without losing global redox control [27].

Emerging evidence further suggests that different peroxiredoxin isoforms perform distinct functions throughout the cell cycle. PRX1 has been implicated in regulating centrosome duplication and mitotic spindle integrity [27]. During early mitosis, Cyclin B–CDK1 phosphorylates PRXI at Thr90, reducing its peroxidase activity by approximately 80% [83,84]. This reversible inactivation allows localized accumulation of H_2_O_2_ around centrosomes, resulting in oxidation and inhibition of the phosphatase Cdc14B. Suppression of Cdc14B delays activation of APC/C–Cdh1, thereby stabilizing Cyclin B1, Aurora A, and Plk1, all of which are essential for mitotic entry [39,85]. Following completion of mitosis, PRXI activity is restored, peroxide levels decline, and normal phosphatase activity resumes [86,87]. These studies indicate that PRXI may be acting as a molecular switch coordinating centrosomal H_2_O_2_ signaling with mitotic progression. Nuclear PRX2 promotes activation of the JNK/c-Jun pathway following genotoxic stress, facilitating efficient DNA repair and recovery from checkpoint activation [88]. Loss of mitochondrial PRX3 disrupts mitochondrial morphology, increases peroxide accumulation, impairs mitochondrial dynamics, and induces G2/M arrest, demonstrating that mitochondrial peroxide signaling is tightly coupled to nuclear cell-cycle progression [89]. Meanwhile, PRX4 within the endoplasmic reticulum integrates oxidative protein folding with intracellular stress responses [90]. PRXVI differs from other family members because it possesses both glutathione peroxidase and phospholipase A_2_ activities [91]. Deletion of PRXVI induces mitochondrial dysfunction, metabolic reprogramming, oxidation of proliferating cell nuclear antigen (PCNA), disruption of glutathione/glutaredoxin signaling, and G2/M arrest [92].

The biological functions of peroxiredoxins are highly context-dependent. In normal cells, PRXs maintain genomic integrity by preventing excess peroxide accumulation, and transient PRX inactivation facilitates localized peroxide signaling that promotes cell-cycle progression. In contrast, many cancers overexpress specific PRX isoforms to tolerate elevated oxidative stress associated with oncogenic transformation and metabolic reprogramming [93,94]. Conversely, genetic deletion or pharmacological inhibition of individual PRXs impairs proliferation, disrupts mitochondrial function, induces G2/M arrest, and sensitizes tumor cells to chemotherapy [94,95]. Thus, peroxiredoxins may function either as tumor suppressors by preserving genome stability or as tumor-promoting proteins by enhancing the antioxidant capacity of established cancers.

**Figure 2:**
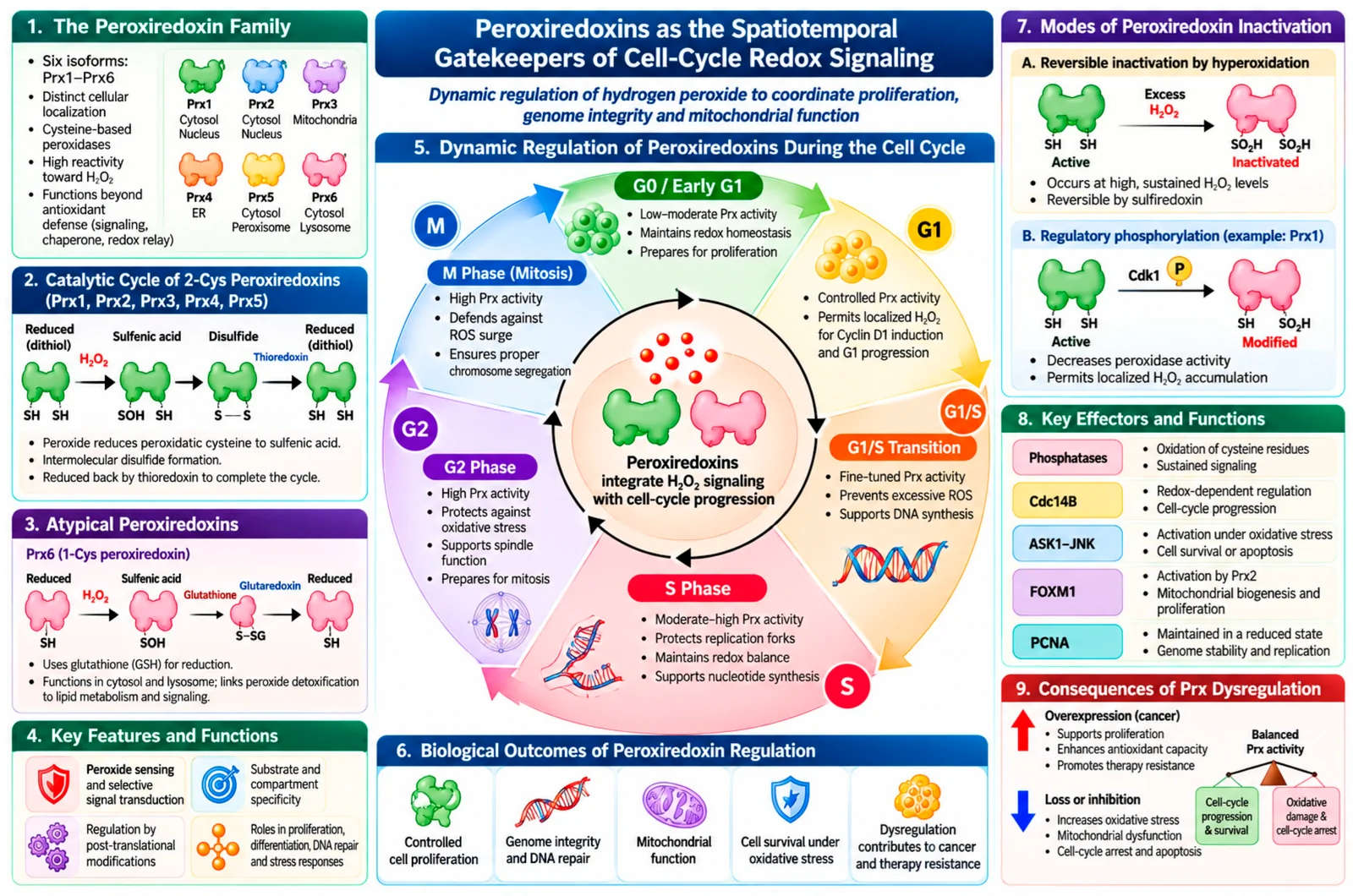
Peroxide sensing and buffering during cell-cycle progression. (Different colors distinguish cell-cycle phases and functional modules for visual clarity and do not represent quantitative differences.).

PRX proteins function as dynamic regulators of H_2_O_2_ signaling rather than constitutive peroxide scavengers. Current evidence establishes peroxiredoxins as considerably more than antioxidant enzymes. They function as the principal interpreters of hydrogen peroxide-dependent signaling, converting a broadly distributed oxidant into highly selective biological information. Recent advances in live-cell imaging and genetically encoded redox biosensors have provided experimental evidence for spatially restricted and temporally dynamic H_2_O_2_ signaling within cells [96]. These observations support the concept that H_2_O_2_ signaling is organized in a compartment- and time-dependent manner; however, the extent and functional significance of this organization may vary among cellular contexts (Figure 2).

### 3.4. Nuclear Factor Erythroid 2-Related Factor 2 (NRF2): The Transcriptional Integrator of the Cellular Redox Program

#### 3.4.1. NRF2 as Cell-Cycle Regulator

NRF2 has emerged as an important regulator of cell proliferation and cell-cycle progression, linking cellular redox homeostasis and metabolism with pathways that control cell growth [97,98,99,100]. Experimental studies have shown that NRF2 deficiency can impair cell-cycle progression through disruption of GSH-dependent redox signaling, while NRF2-dependent metabolic reprogramming can increase NADPH generation, glutathione synthesis, and anabolic metabolism in proliferating cells [30]. NRF2 can also participate directly in the DNA-damage response through ATR–CHK1–CDC2 signaling and G2 arrest, demonstrating that its role extends beyond antioxidant transcription [31].

In synchronized cell populations, NRF2 protein accumulates at the G1/S and early S phases, decreases in G2, and reaches its minimum value in mitosis [29,101]. These oscillations are also reproduced in the NRF2 ablation-induced cell accumulation in the phases G1 and M, thus suggesting that NRF2 activity is indispensable for passing through different checkpoints of this process. In these respects, NRF2 acts differently with peroxide and peroxiredoxin signaling. Peroxide-mediated redox signaling can occur on a time scale of seconds to minutes through reversible oxidation of redox-sensitive cysteine residues in target proteins [102,103]. By contrast, NRF2 executes long-term adaptation by altering gene expression carefully over hours to days. For example, NRF2 may first have to be activated so that it can meet a considerable metabolic challenge at the G1/S transition and prepare the cell for DNA replication. NRF2 thus activates an elaborate network of cytoprotective genes: MnSOD, catalase, enzymes responsible for glutathione biosynthesis and glutathione peroxidases, thioredoxin and thioredoxin reductase enzymes, NAD(P)H quinone oxidoreductase 1 (NQO1), heme oxygenase-1 (HO-1), peroxiredoxins, and many enzymes involved in NADPH regeneration [104]. Highly proliferating cells require nutrients; energy; and excessive synthesis of nucleotides, amino acids, and lipids. NRF2 upregulates the pentose phosphate pathway, glutamine utilization, and NADPH production, supporting the synthesis of bioconstituents [105,106] At the G1/S transition, an intracellularly highly reducing microenvironment is needed to facilitate nucleotide production, protein synthesis, mitochondrial metabolism, and DNA replication and protect the cell against a sudden increase in oxidative stress. NRF2 induces a highly protective cellular redox microenvironment through the transcriptional activation of genes involved in glutathione biosynthesis, thioredoxin metabolism, NADPH synthesis, and other antioxidant defenses [105]. Studies in cancer cells and glioma stem cells indicate that NRF2 loss can impair the G1/S transition. This phenotype is associated with reduced Cyclin E expression and CDK2 activity, decreased Rb phosphorylation, and suppression of E2F-dependent transcription, collectively limiting progression into the S phase [30]. The principal cell-cycle and DNA-damage targets associated with NRF2 are summarized in Table 3. NRF2 also transcriptionally regulates RAD51, which is implicated in homologous recombination and replication-associated DNA repair [31].

After a DNA double-strand break, NRF2 was immediately recruited to the damage site and interacted with ATR to activate the ATR–CHK1–CDK1 signaling cascade, thus inducing transient G2 arrest, promoting homologous recombination, and preserving genomic integrity [31]. Experimental depletion or knockout of NRF2 impairs ATR–CHK1 signaling and G2 checkpoint activation following DNA damage, resulting in increased cellular sensitivity to ionizing radiation [31]. Second, the G2/M progression via glutathione-dependent redox signaling depends on NRF2; genetically deleted, NRF2 promotes glutathione depletion and oxidative damage to DNA, resulting in a long-lasting G2/M arrest [30]. Restored intracellular glutathione, but not mere ROS scavenging by N-acetylcysteine, rescues normal cell-cycle progression through the reactivation of Akt signaling and relocalization of mitosis-promoting proteins [45,107,108]. During maturation in the mouse oocyte, NRF2 was dynamically localized from the germinal vesicle to spindle microtubules [109]. Depletion of NRF2 perturbed spindle assembly and chromosome alignment during meiotic progression and Cyclin B1 expression [109]. These support a possible role for NRF2 in spindle organization and chromosome segregation during the late cell-division stages, indicating that NRF2 may act as another player in any checkpoint regulation level.

The other genomic and epigenomic studies, for instance, the presence of cross-talk between NRF2 and other central regulators, such as p53, NF-κB, HIF-1α, MYC, transcription factors from the FOXO family, and various chromatin-remodeling complexes, revealed the vast regulatory landscape of NRF2 [110,111,112,113,114]. Integration of NRF2 into the oxidative stress response, inflammatory signaling, nutrient sensing, and DNA damage checkpoint continuum allows the cell to weigh proliferative choices against metabolic and redox needs. Such cross-talk further corroborates the resistance to any attempt to view cell-cycle control mechanisms through a single signaling pathway lens since their modulation depends on multiple interactions across several signaling pathways.

#### 3.4.2. Context-Dependent Role of NRF2

**Figure 3:**
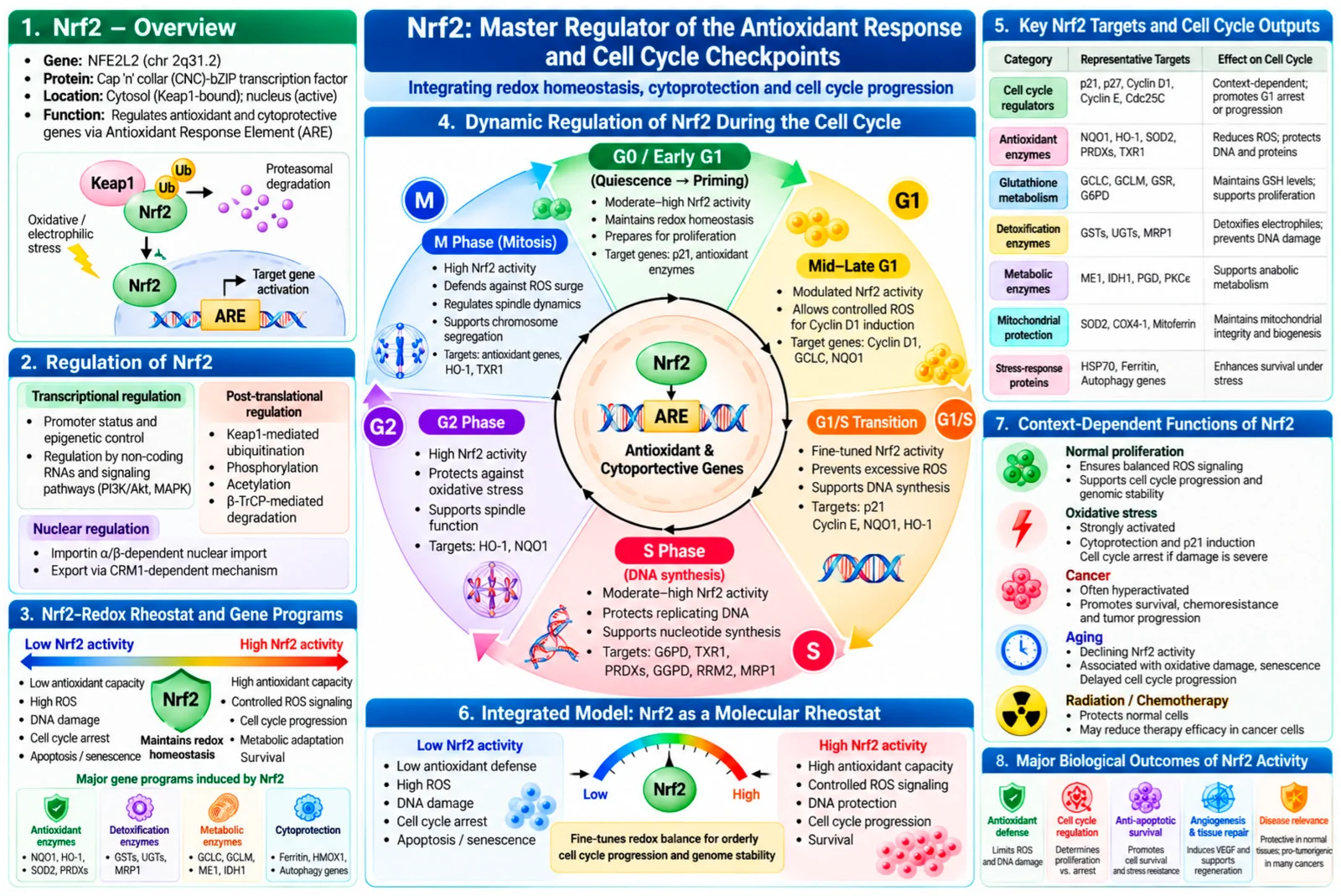
Metabolic adaptation and phase-specific cell-cycle regulation by NRF2. (Different colors distinguish cell-cycle phases and functional modules for visual clarity and do not represent quantitative differences.)

The effects of NRF2 are highly context-dependent. NRF2 activation within physiological proliferation levels drives the pro-cell-cycle progression processes responsible for intracellular reducing conditions driving the active states of cyclin–CDK complexes, DNA synthesis, and mitotic spindle progression. Under conditions of oxidative or electrophilic stress, NRF2 acts as a checkpoint regulator via the PERK-NRF2 pathway, inducing the cyclin-dependent kinase inhibitors p15, p21, and p27, thereby suppressing cyclin D1-CDK4/6 activity and inducing transient G1 arrest [115,116,117]. Under mild genotoxic stress, protective p16INK4a expression through NRF2 suppresses Rb phosphorylation and prevents inappropriate entry into the S phase due to DNA-damage accumulation [118]. These observations explain how, depending on the degree and type of intracellular stress, NRF2 activity may promote or counteract proliferation (Figure 3).

### 3.5. Heme Oxygenase-1 (HO-1): A Metabolic Redox Switch Between Proliferation and Stress Adaptation

#### 3.5.1. HO-1 in Cell-Cycle Regulation

Heme oxygenase-1 (HO-1) is one of the most dynamic stress-induced redox regulatory proteins described so far for cell-cycle control. Unlike constitutive antioxidant enzymes and proteins, HO-1 is strongly induced under oxidative stress, proinflammatory cytokines, hypoxia, irradiation, heavy metals, hyperglycemia, heme, and electrophiles, mainly through the activation of the NRF2 signaling pathway [119,120]. In that respect, HO-1 catabolizes pro-oxidant heme to carbon monoxide, biliverdin/bilirubin, and ferrous iron, thus eliminating an oxidant while generating biologically active metabolites that regulate intracellular signaling [121]. The generally accepted notion that HO-1 is a cytoprotective stress protein emerged due to its great inducibility and the apparent fact that HO-1 expression protects cells against oxidative injuries. However, some of the most direct evidence for HO-1 in regulating cell-cycle progression was originally reported by Abraham’s group in endothelial cells [122]. Indeed, the group first reported, in 2002, that HO-1 overexpression prevented abnormal oxidative DNA distribution associated with G2/M accumulation, an effect reversed by the HO activity inhibitor 6-aminonicotinamide [123,124,125]. Follow-up studies by that group showed that HO-1 overexpression increased endothelial proliferation, decreased p21 and p27, and changed the expression pattern of many genes related to growth [124,125]. Such a rapid development has thus turned the biology of HO-1 from that of a pure cytoprotective protein to that involved in the coordination of cellular recovery with proliferative capacity.

Although HO-1 regulates several cell-cycle components, the direction of this regulation depends on the type of cells and physiological conditions. HO-1 induces Cyclin D1 and Cyclin E, vascular endothelial growth factor (VEGF), EGF, and HDGF in proliferative endothelial cells, favoring proliferation and angiogenesis [126,127,128]. In contrast, HO-1 pharmacological inhibition in thyroid carcinoma decreases Cyclin D1 and CDK4 expression while increasing p21 and p27; thus, growth arrest is finally achieved [35]. This thus suggests that HO-1 acts mainly upstream of the proliferation signal but not directly on the transcription of cyclin genes.

Colombrita et al. found that, in quiescent endothelial cells, angiotensin II induced HO-1 already at the G0/G1 phase, before DNA synthesis takes place—an induction that acts to reduce oxidative damage to DNA [129]. In quiescent cells, the induction of HO-1 by the prooxidants heme and angiotensin II in G0/G1 favors an intracellular environment for redistributing metabolism upon exiting the cell cycle. Bilirubin, an HO-1 product, is highly efficient in scavenging reactive oxygen species; inhibitor of HO activity dramatically enhances oxidative damage to DNA and impairs progression through this early phase of G1 [124].

#### 3.5.2. Context-Dependent Functions of H_2_O_2_

The effects of HO-1 on the G1/S transition are complex and vary with different biological contexts. In normal endothelial cells, HO-1 promotes progression in G1 from inhibition of p21 and p27 and increased expression of Cyclin D1, Cyclin E, VEGF, EGF, and HDGF, which facilitate DNA synthesis and cell proliferation [126,127,128]. However, ectopic HO-1 expression in pulmonary epithelial cells delays G1/S transition with an increment in cells at G0/G1 and suppression of DNA synthesis [130]. These contrary effects suggest that HO-1 does not always stimulate proliferation, but according to intracellular redox, HO-1 may allow cells to replicate their DNA by temporarily arresting cells in the S phase.

Although only a few studies on the effects of HO-1 in the S phase are available, HO-1 is indirectly positive for DNA replication because it maintains genome integrity. In this way, HO-1 favors proper DNA replication through the reduction of intracellular ROS and oxidative DNA damage. On the other hand, chronic oxidative stress and long-lasting HO-1 expression positively prevent entry into S phase, giving cells more time to repair before replication. Hence, HO-1 modulates a balance in proliferation vs. genome protection instead of acting on DNA synthesis.

It is, therefore, implicated in G2/M, although cellular context determines whether the effects are positive or negative in that cell-cycle phase. In this regard, the originally reported work by Kushida et al. showed that overexpression of HO-1 reverses oxidative stress-induced aberrations associated with accumulation in G2/M [131]. Again, CO was postulated as a significant mediator of this effect. In contrast, in esophageal squamous carcinoma cells, HO-1 induces G2/M arrest yet confers resistance to apoptosis through activation of the p38 MAPK and mTOR pathways [132]. Such diverse outcomes have seemingly weighed HO-1 during various phases of the cell cycle-like cyclins and CDKs.

Indeed, HO-1 activity would be context-specific. In this setting, the transient induction of HO-1 under normal conditions leads to increased survival, angiogenesis, and cellular proliferation with the concomitant suppression of oxidative DNA damage. Under hyperglycemic conditions, HO-1 favors cell-cycle progression by dampening p21 and p27 levels; however, further or chronic induction of HO-1 may transiently inhibit transition into S phase or may be a proliferative transition state [125]. On the other hand, most tumors have already adapted to high HO-1 expression and thus escape oxidative stress and antitumor treatment. The inhibition of HO-1 was associated with G0/G1 arrest, downregulation of Cyclin D1 and CDK4, upregulation of p21 and p27, and increased intracellular ROS, with a marked reduction in tumor growth in thyroid cancer [35]. The available literature is suggestive of an alternative role of HO-1 during cell-cycle commitment decisions.

**Figure 4:**
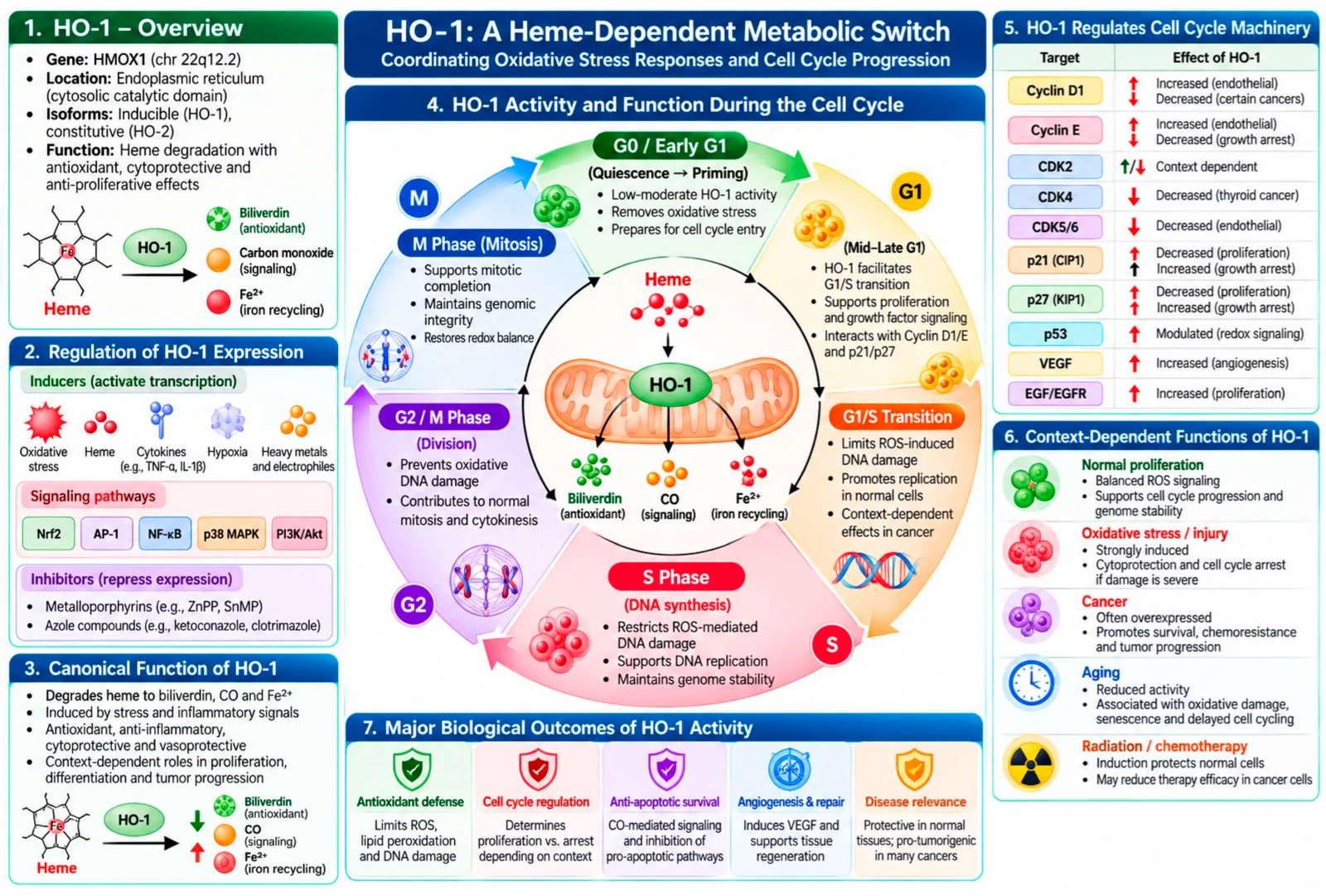
Integration of heme metabolism, redox signaling, and cell cycle. (Different colors distinguish cell-cycle phases and functional modules for visual clarity and do not represent quantitative differences).

We propose that HO-1 may functions as a metabolic redox switch, converting heme stress into context-dependent signals that align proliferative commitment with the cell’s capacity to tolerate and repair oxidative injury (Figure 4).

### 3.6. NAD(P)H:Quinone Oxidoreductase 1 (NQO1) and Cell-Cycle Progression

NAD(P)H:quinone oxidoreductase 1 (NQO1), originally identified as a phase II detoxification enzyme, catalyzes the two-electron reduction of quinones to hydroquinones, thereby preventing semiquinone formation and redox cycling [38,133]. It is becoming increasingly known that NQO1 performs numerous non-canonical functions extending beyond xenobiotic metabolism and plays role in regulation of the NAD(P)H/NAD(P)^+^ ratio, stabilization of proteins, modulation of mRNA translation, and interaction with mitotic spindle components.

Studies by Shaul and colleagues revealed that NQO1 stabilizes p53 and p73, and later work extended this to include p33ING1b, C/EBPα, HIF-1α, and other regulatory factors [134,135,136].

The first direct links between NQO1 and proliferation emanated from cancer-cell models, wherein Garate and colleagues demonstrated that NQO1 promotes cell-cycle progression and increases the expression of Cyclins D1 and A2 in melanoma cells [137]. NQO1 increased the stability of nuclear BCL3, and it supported NF-κB p50 signaling and, consequently, melanoma-cell proliferation. NQO1 depletion reduced the half-life of BCL3 to suppress proliferation. Similarly, NQO1 knockdown caused accumulation of cells in G1, reduced the S-phase population and Cyclin D1, and increased p21 in cholangiocarcinoma.

NQO1 maintains reducing environment while stabilizing proteins required for proliferation, which helps in G1/S transition. Further, increased NQO1 expression enhances Cyclin D1 and Cyclin A2 levels, allowing S-phase entry, whereas NQO1 depletion delays DNA synthesis and reduces clonogenic growth [137]. Interestingly, while NQO1 promotes Cyclin D1-dependent proliferation, it also stabilizes p53 family members that are known to induce cell-cycle arrest [134,138]. These findings point that NQO1 may not be a sole determinant of the proliferative or resting phase of cells; rather, it preserves the regulatory proteins required for the cell to make that decision. Stabilization of BCL3 and other growth-associated proteins supports progression during favorable conditions, whereas, following oxidative or genotoxic stress, stabilization of p53-family proteins reinforces checkpoint activation.

Existing evidence clearly suggests that NQO1 contributes to genome stability by maintaining NAD(P)H-dependent redox homeostasis and preventing quinone-mediated oxidative stress. NQO1 minimizes oxidative DNA damage that could compromise DNA replication via stabilization of p53 proteins [138].

A rather unexpected finding from Siegel and colleagues has shown that NQO1 localizes to the mitotic spindle [139]. Subsequent work by Kang et al. demonstrated that NQO1 colocalizes and directly interacts with SIRT2 during mitosis [37]. NQO1 supports SIRT2 activity by providing NAD^+^, thereby influencing the acetylation state and assembly of the anaphase-promoting complex/cyclosome (APC/C). NQO1 depletion perturbs APC/C function and delays mitotic progression, while NAD^+^ precursor supplementation can alleviate aspects of the mitotic defect. This work provides a mechanistic link between pyridine nucleotide metabolism, redox enzyme activity, and the machinery controlling mitotic exit.

In 2023, studies demonstrated that NQO1 physically interacts with the DNA-binding domain of c-Fos and protects it from proteasomal degradation [36]. Subsequently, stabilized c-Fos promotes CKS1 expression that in turn provides support to CDK1 activity, allowing G2/M progression. Loss of NQO1, however, reduces c-Fos and CKS1, resulting in delayed progression through G2/M and altered response of cancer cells to ionizing radiation. NQO1 functions diversely and performs quinone reduction, p53 stabilization, BCL3 regulation, spindle localization, SIRT2 activation, and c-Fos protection, which seems to be scattered functionality. However, what appears to be a common mediator is redox-dependent protein stability and protein function. These established interactions place NQO1 among the redox regulators with defined links to cell-cycle regulatory proteins (Table 3).

**Figure 5:**
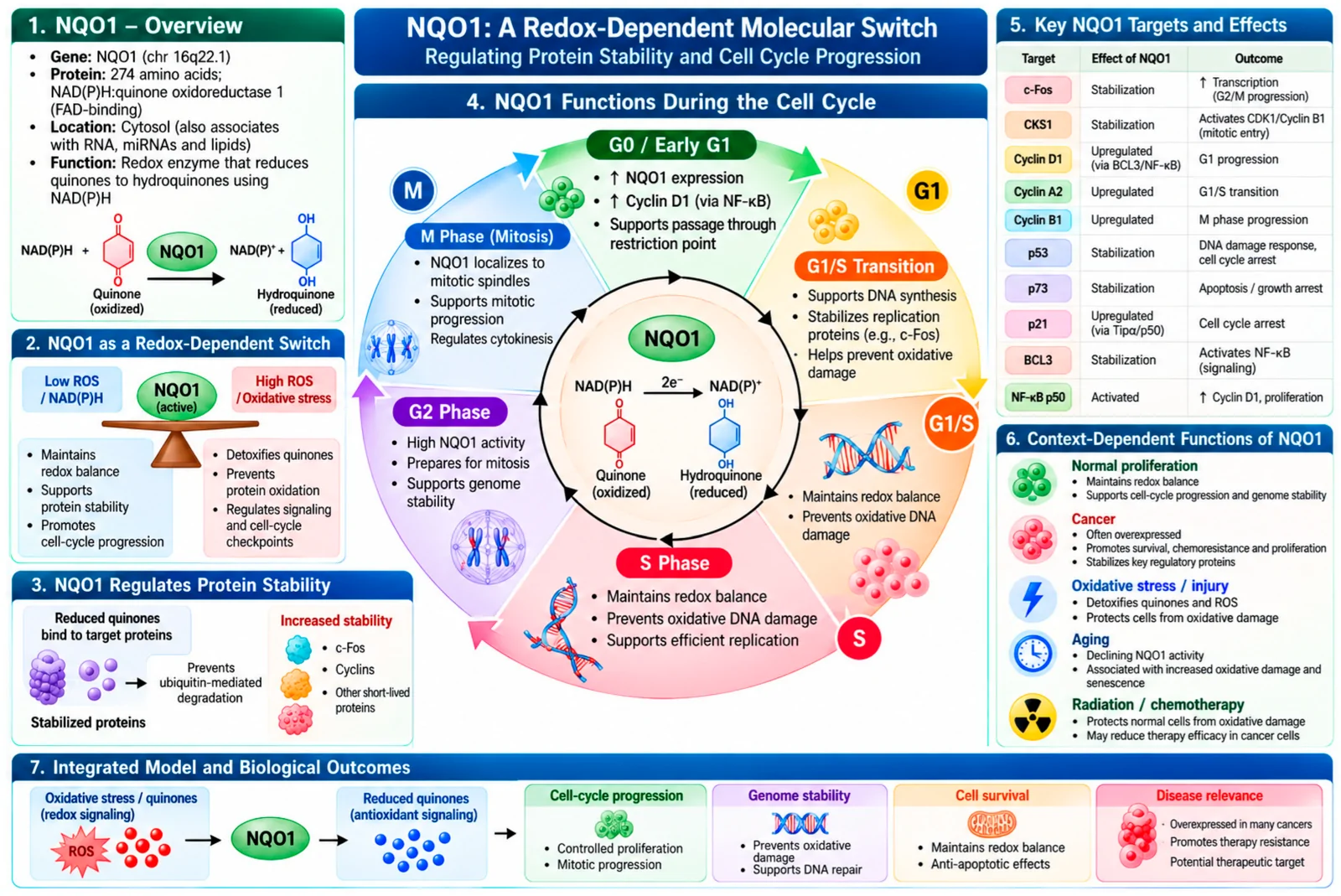
A redox switch translating NADPH currency in cell cycle. (Different colors distinguish cell-cycle phases and functional modules for visual clarity and do not represent quantitative differences.).

Interestingly, it appears that NQO1 couples the NAD(P)H-dependent redox state to the stability and activity of proteins that are involved in one way or other in cell-cycle regulation (Figure 5).

### 3.7. Glutathione: Spatial Redistribution of the Cellular Redox Buffer During Cell-Cycle Progression

Glutathione, the most abundant tri-peptide antioxidant is maintained at millimolar concentrations in most cells and supplies reducing equivalents to glutathione peroxidases, glutaredoxins, and glutathione S-transferases. It participates in reversible protein S-glutathionylation and the dismutation of free radicals, and it contributes to the maintenance of thiol groups in a reduced state. For several years, this was the known primary function of GSH.

Early studies revealed that depleting the cellular pool of GSH inhibits cell growth and arrests cells before DNA synthesis, while restoration of the GSH pool facilitates progression from G1 into S phase [44]. Oxidation of glutathione is associated with impaired cell-cycle progression, suggesting that glutathione is needed during proliferation. This is easily conceivable, as proliferation increases oxidative metabolism and GSH protects cells against the resulting oxidative burden.

The work of Pallardó, Viña, and colleagues, however, introduced a different perspective about the role of nuclear glutathione in cell cycles [43]. Using confocal analysis of glutathione distribution in proliferating fibroblasts, Markovic et al. demonstrated that GSH accumulates within the nucleus during the early phases of cell proliferation [140]. The nuclear-to-cytoplasmic GSH ratio peaked following the plating of cells and remained elevated during exponential growth, when a substantial proportion of cells were progressing through S and G2/M. As cells reached confluence and accumulated in G0/G1, this asymmetric distribution disappeared, and GSH returned to a predominantly cytoplasmic and perinuclear pattern. These observations suggested that the relationship between glutathione and the cell cycle is governed not simply by the amount of GSH present but also by where the reducing capacity is positioned within the cell.

GSH associates closely with nuclear DNA during proliferation, and the nuclear glutathione pool is implicated in DNA synthesis, chromatin regulation, and DNA repair. In addition, glutathione influences transcriptional regulation and chromatin organization through reversible thiol modifications of nuclear proteins, thereby contributing to gene expression and maintenance of chromatin architecture during rounds of cell division [141,142]. Subsequent to nuclear envelope dissolution during mitosis, nuclear and cytoplasmic pools equilibrate before glutathione is redistributed between daughter cells. More recent analyses continue to support a dynamic nuclear GSH pool; however, whether it is maintained independently from cytosolic glutathione or equilibrates through nuclear pores remains unresolved [143].

Protein S-glutathionylation adds a second regulatory dimension to this system, which is discussed under the glutaredoxin section.

Additionally, in normal tissues, maintenance of adequate GSH levels preserves genome integrity, supports DNA replication, and protects proliferating cells from oxidative injury, but many cancers exhibit increased glutathione synthesis and enhanced glutathione-dependent metabolism to overcome therapy-associated oxidative stress.

We propose that glutathione functions as a mobile redox buffer that partitions reducing power across intracellular compartments, allowing cytoplasmic redox signaling to coexist with nuclear genome protection during cell-cycle progression.

### 3.8. Glutathione Peroxidases: Setting the Peroxide Threshold for Proliferative Redox Signaling

Use of glutathione as a reducing currency and oxidative stress checkpoint is possible because of glutathione peroxidase (GPX), which catalyzes the oxidation of GSH to GSSG and, in the process, neutralize peroxides. As discussed earlier, considering the widespread distribution of peroxide microdomains, in order to regulate site-specific peroxides levels, eight GPX family members, GPX1–GPX8, operate at distinct subcellular locations that have varied catalytic characteristics and substrate preferences. GPX1 is widely distributed in the cytoplasm and mitochondria, GPX2 is enriched in rapidly renewing epithelial tissues, GPX3 functions predominantly in extracellular environments, and GPX4 possesses the unusual ability to reduce phospholipid hydroperoxides within biological membranes. GPX7 and GPX8 are associated primarily with the endoplasmic reticulum and oxidative protein folding [41,144]. The very fact that cells require multiple spatially and biochemically distinct enzymes suggests that their function is not simply to remove peroxides from the system but rather to function as a regulatory process. Consistent with our earlier discussion about the role of H_2_O_2_ during cell-cycle progression depending on concentration, localization, and duration, it is perceivable that cells need to design the system to achieve this in a spatiotemporal manner. GPXs therefore operate to prevent peroxide concentrations from becoming damaging without extinguishing the oxidative signals required for proliferation. In this respect, their role differs conceptually from that of peroxiredoxins. Prxs can function as highly sensitive peroxide sensors and redox relays, whereas the GPX system contributes substantially to buffering the peroxide environment in which those signals operate.

Current evidence indicates that GPXs regulate cell-cycle progression predominantly through modulation of intracellular peroxide tone rather than direct interaction with cyclins or cyclin-dependent kinases [28,145]. By controlling peroxide availability, GPXs indirectly influence multiple signaling pathways involved in proliferation, cell-cycle regulation, metabolism, and genome integrity viz. Cyclin D1, Cyclin A, AMPK, ERK1/2, mTOR, NRF2 pathway signaling, DNA damage signaling, and also the GPX4-ferroptosis axis [28,144,146]. One thing that can be noted here is that, rather than acting as classical cell-cycle proteins, GPXs function to shape the cellular redox environment, which then determine cell-cycle progression.

Early studies showed that overexpression of GPX4 prolonged cellular doubling time and delayed G1/S progression by reducing phospholipid hydroperoxides required for proliferative signaling [145]. Similarly, GPX3 overexpression induced G0/G1 arrest through modulation of AMPK and ERK1/2 signaling, whereas GPX3 deficiency restored proliferative capacity [147]. It seems a bit counterintuitive, as higher antioxidant capacity should have been conducive to cell proliferation. However, if we view through the framework of redox signaling, it appears logical that reducing the peroxide signaling below threshold may not lead to reversibly oxidized growth-regulatory proteins, leading to blocking traversal through the restriction point.

**Figure 6:**
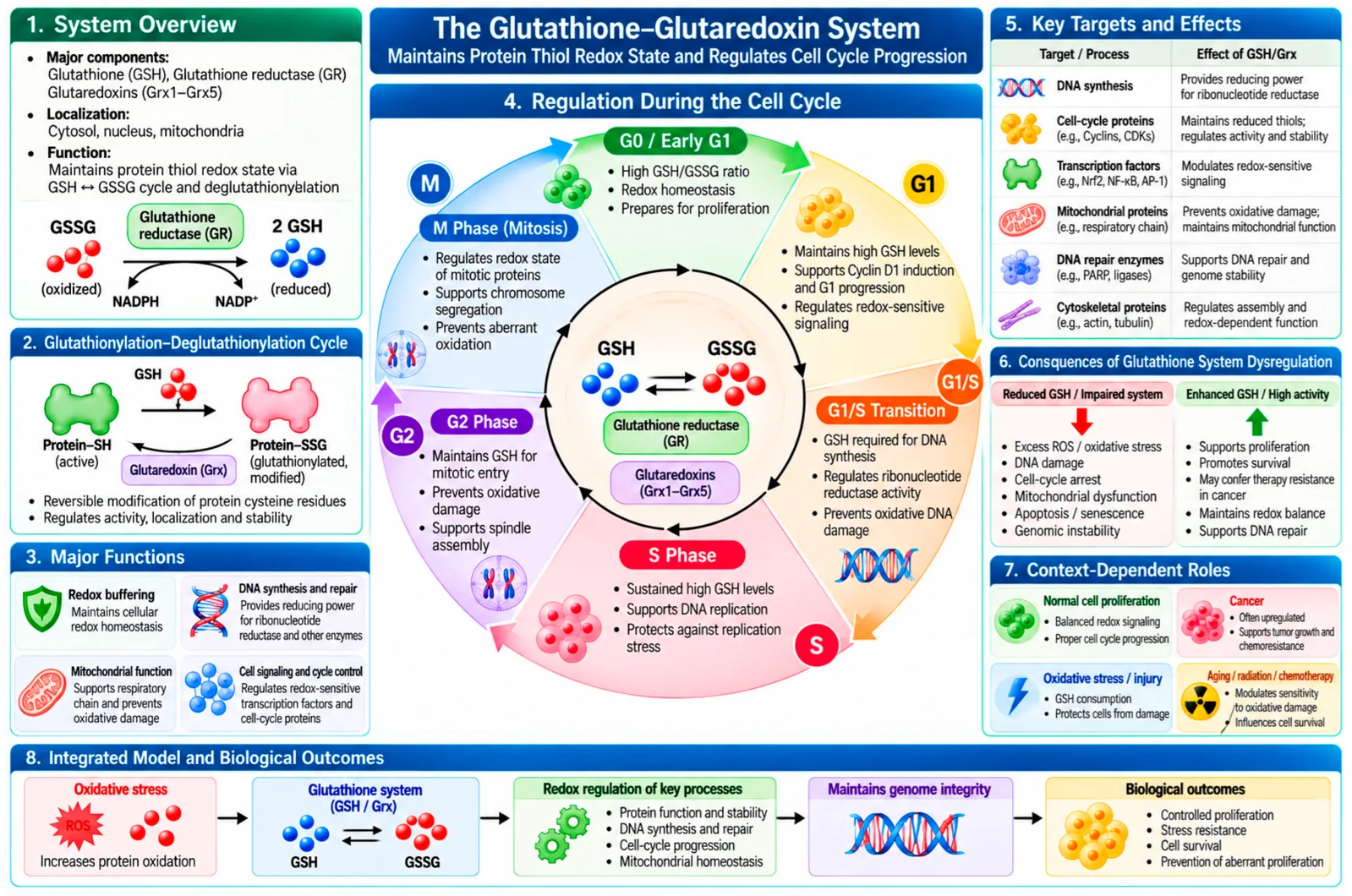
Glutathione and glutaredoxin networks in cell-cycle regulation. (Different colors distinguish cell-cycle phases and functional modules for visual clarity and do not represent quantitative differences.).

It is reported that human-induced pluripotent stem cells express high levels of GPX2, together with elevated glutathione, enabling efficient protection against mitochondrial and nuclear DNA damage [42]. Depletion of GPX2 markedly increases oxidative DNA lesions, demonstrating that the glutathione–GPX system safeguards DNA replication by limiting oxidative injury while maintaining physiological redox signaling compatible with S-phase progression. GPX4 acts on phospholipid hydroperoxides embedded within biological membranes to prevent uncontrolled phospholipid peroxidation, and it preserves membrane integrity to evade induction of ferroptosis [148]. We propose that glutathione peroxidases function as threshold setters within the cellular Redox Code. Their contribution to cell-cycle progression is therefore not to drive a particular phase directly, but to determine whether the oxidative environment remains within the range in which proliferation is biologically possible (Figure 6).

### 3.9. Glutaredoxins: Restoring Reversibility to Protein Redox Signaling During Cell-Cycle Progression

Glutaredoxins (Grxs) catalyze the glutathione-dependent reversible protein S-glutathionylation, thus distinguishing them from most of the antioxidant proteins discussed earlier [149]. Till now, we have seen that MnSOD changes the chemical identity of mitochondrial ROS, peroxiredoxins operate in the microdomains of peroxide signaling, and GPXs establish peroxide thresholds. Interestingly, glutaredoxins operate at a level where the oxidative information has reached protein thiols. By catalyzing deglutathionylation, they determine the lifetime of a redox-dependent protein modification and restore susceptible proteins to a reduced functional state [150]. It appears to be the connecting link between cellular glutathione pool and reversible regulation of protein activity via thiol-disulfide exchange.

Among several isoforms, Grx3 has provided some of the most direct evidence linking glutaredoxins to mammalian cell-cycle progression. Genetic deletion of Grx3 causes embryonic lethality, while Grx3-deficient embryonic fibroblasts exhibit impaired proliferation and defective progression through G2/M [151]. Grx3-deficient cells show delayed mitotic exit and accumulation of multinucleated cells. Similar observations following Grx3 depletion in HeLa cells support a requirement for Grx3 during successful completion of mitosis and cytokinesis. The Grx3 studies suggest that reversible thiol regulation remains equally important after DNA replication has been completed. Mitosis requires coordinated remodeling of the cytoskeleton, spindle apparatus, protein complexes, and intracellular membranes. Many proteins participating in these events contain redox-sensitive cysteines. Although the precise Grx3 substrates responsible for the mitotic phenotype remain incompletely defined, the accumulation of multinucleated cells following Grx3 loss suggests that failure to appropriately resolve redox-dependent protein modifications can disrupt the final stages of cell division.

If we look at the available evidence in the literature, it is shown that, in human lens, epithelial-cell decrease in Grx1 leads to enhanced ROS, oxidation of glutathione followed by activation of ERK activation, and subsequently G1 arrest and apoptosis [152,153]. On the contrary, overexpression of Grx1 restores redox balance and inhibits sustained ERK activation, leading to alleviation of G1 arrest. Interestingly, Grx1 is also known to reduce selected mammalian RNR, which is responsible for de novo deoxyribonucleotide synthesis through the glutathione-dependent pathway [47].

Through cycles of glutathionylation and deglutathionylation, glutaredoxins ensure that redox signals are reversible and help in protecting critical proteins against irreversible oxidation. This specialized function distinguishes glutaredoxins from other antioxidant systems and places them at the interface between glutathione homeostasis and redox signaling. Their role in cell cycle is shown in Figure 6.

It is reported that loss of glutaredoxin activity impairs cell division and promotes checkpoint activation [48,154]. Many cancers exploit elevated glutaredoxin expression to maintain redox homeostasis during the periods of oxidative stress in the process of rapid proliferation [47]. Within the cell cycle, their contribution may be understood as restoring redox-regulated proteins after each oxidative instruction has been executed, thereby allowing the next phase of proliferative signaling to proceed.

### 3.10. Thioredoxin/Thioredoxin Reductase: The Reductive Engine Coupling Cellular Metabolism to DNA Replication

#### 3.10.1. Thioredoxin System and Cell Cycle

The thioredoxin (Trx) system consists of NADPH, thioredoxin reductase (TrxR), and thioredoxin, which together act to reduce oxidized proteins via reduction of disulfide bonds [155]. TrxR is a selenoenzyme that transfers electrons from NADPH to the conserved dithiol active site of thioredoxin, thereby reducing it. Reduced Trx function restores oxidized cysteine residues within target proteins [156]. The TrxR/Trx system functions in tandem to reduce several proteins containing oxidized cysteine residues, including transcription factors NRF2, NF-κB, p53, and APE1, which, in one way or another, regulate cellular metabolism critical to cell-cycle progression [157,158]. Other than this, one of the prominent roles of the TrxR/Trx system is to reduce ribonucleotide reductase, the only enzyme capable of converting rNTPs into dNTPs, which are critical for DNA synthesis [49]. Therefore, there is a direct biochemical regulation of this process of DNA synthesis that happens during the S phase of the cell cycle.

Mammalian cells maintain two major thioredoxin systems, cytosolic TRX1 and TRXR1, and mitochondrial TRX2 and TRXR2. [159] However, it is noteworthy that TRX1 is not confined to the cytoplasm; under proliferative and stress conditions, it can translocate to the nucleus, where it influences redox-sensitive transcription factors and nuclear proteins. TRX2, in contrast, supports mitochondrial redox control and works closely with mitochondrial peroxiredoxin-3. This compartmentalization allows the thioredoxin system to connect cytoplasmic metabolism, nuclear DNA synthesis, and mitochondrial function. In addition to reducing peroxiredoxins, thioredoxin regulates numerous redox-sensitive proteins, including apoptosis signal-regulating kinase-1 (ASK1), NF-κB, AP-1, HIF-1α, PTEN, and ribonucleotide reductase (RNR), thereby integrating cellular metabolism with cell-cycle progression [159].

In agreement with the above findings, quiescent cells exhibit relatively low Trx activity, whereas proliferating cells markedly upregulate TRX1 and TRXR1 [50]. Inhibition or loss of TRXR1 can markedly reduce deoxyribonucleotide pools despite continued metabolic activity, leading to replication stress, DNA damage signaling, and S-phase arrest.

Apart from regulating DNA synthesis and transcription factor binding to DNA, reduced thioredoxin binds and inhibits ASK1 under physiological conditions, whereas oxidative stress dissociates this complex, activating JNK and p38 MAPK signaling to promote cell-cycle arrest or apoptosis [160]. Additionally, redox regulation of PTEN links thioredoxin activity to PI3K/AKT signaling, providing another mechanism that coordinates proliferation with cellular metabolism.

Interestingly, mitochondrial TRXR2/TRX2 limits oxidative stress and prevents mitochondrial permeability transition via reduction of peroxiredoxin-3 and regulation of cyclophilin D that prevents cell death [161,162].

#### 3.10.2. Context-Dependent Functions of Thioredoxin System

The biological functions of the thioredoxin system are highly context-dependent. In normal tissues, Trx/TrxR supports genome stability, DNA replication, and checkpoint regulation during physiological proliferation. During immune activation, transient induction of thioredoxin enables rapid clonal expansion by sustaining nucleotide synthesis [50]. In contrast, many cancers exploit persistent Trx and TrxR overexpression to maintain elevated DNA synthesis, suppress apoptosis, and resist chemotherapy and radiotherapy [158]. Consequently, pharmacological inhibition of TrxR disrupts DNA replication, induces S-phase arrest, and promotes apoptosis, highlighting the thioredoxin system as an attractive therapeutic target.

**Figure 7:**
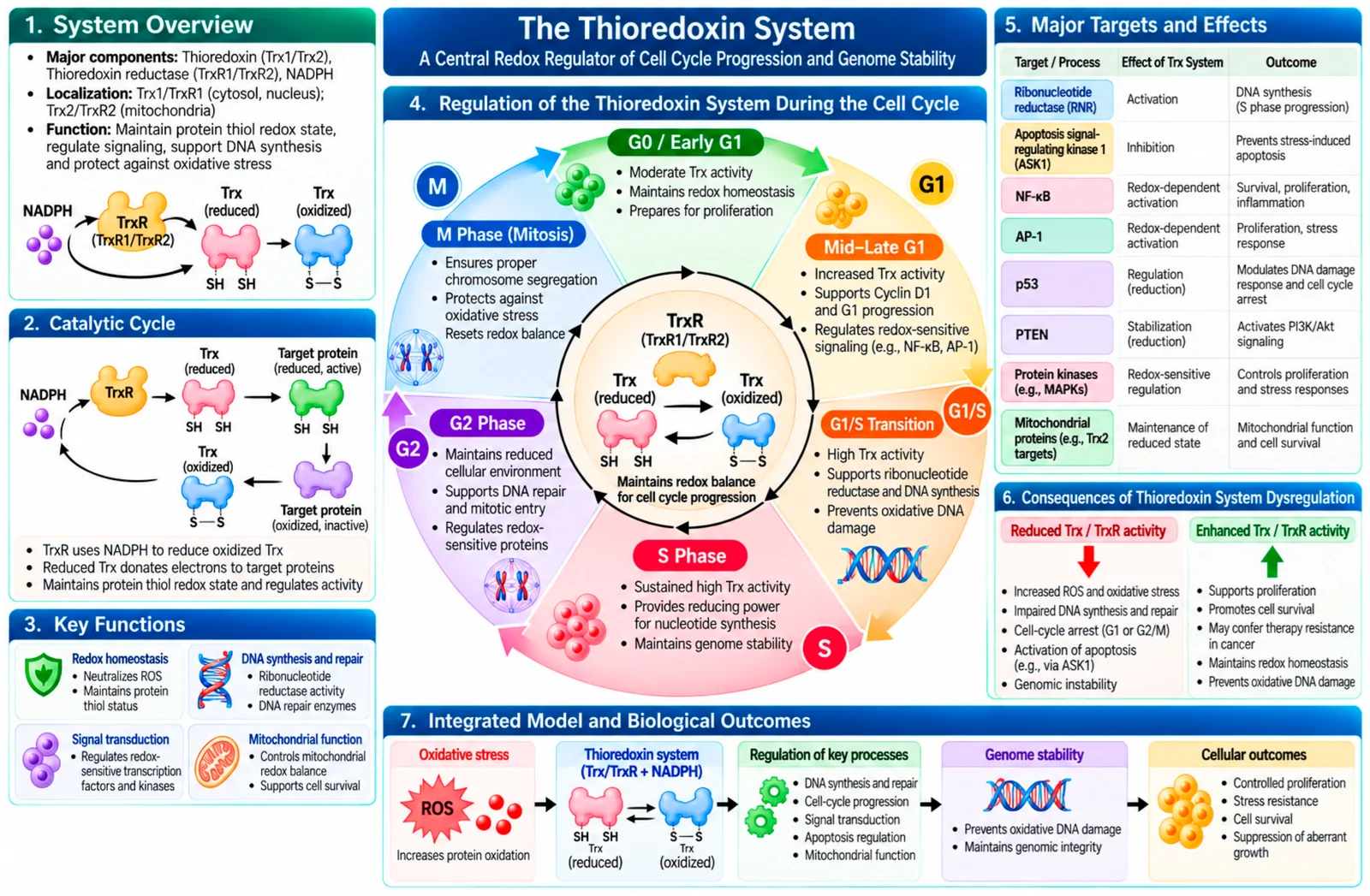
Thioredoxin networks in cell-cycle regulation. (Different colors distinguish cell-cycle phases and functional modules for visual clarity and do not represent quantitative differences.).

The Trx/TrxR system converts NADPH-derived metabolic reducing power into the electrons required to sustain redox-sensitive protein function essential for cellular metabolism and DNA synthesis [163]. A cell may receive every signal required to divide, but without a continuous flow of reducing equivalents through thioredoxin and ribonucleotide reductase, the biochemical execution of genome duplication cannot proceed. In a nutshell, the Trx/TrxR system operates to shuttle electrons and handle the redox sensitive information for cell-cycle regulation. Contributions of both glutathione and thioredoxin systems to cell cycles are shown in Figure 7.

## 4. An Integrated Redox Regulatory Network Governing Cell-Cycle Progression

### 4.1. Cell-Cycle Progression as a Dynamic Redox Process

The integrated analysis of the dynamic changes in redox regulatory systems from the perspective of cell-cycle phases revealed that they are not functioning simply as scavengers of reactive oxygen species (ROS). Rather, these redox regulatory systems operate in a spatio-temporally coordinated manner to relay redox signals regulating cell cycle. One fact that becomes evident upon careful perusal of the existing literature is that oscillations in intracellular oxidation–reduction status function alongside cyclin–CDK signaling to regulate DNA replication, checkpoint activation, chromosome segregation, and cytokinesis.

### 4.2. G1 Phase: Establishing a Proliferative Redox Environment

Superoxide generated through mitochondrial respiration and NADPH oxidase is converted into H_2_O_2_ by MnSOD, and H_2_O_2_ functions as a second messenger promoting growth factor signaling and Cyclin D–CDK4/6 activation. This peroxide signaling is locally controlled in microdomains via peroxiredoxins by permitting transient H_2_O_2_ accumulation while simultaneously attenuating uncontrolled oxidation. Parallelly, NRF2 activation leads to expression of antioxidant and metabolic genes, including HO-1, NQO1, glutathione biosynthetic enzymes (GCLC and GCLM), and components of the glutathione (GPX, GR, and GRX) and thioredoxin systems (Trx/TrxR), to cope with the increased biosynthetic demands of DNA replication. Enhanced glutathione abundance helps in establishing a reducing intracellular environment that prevents irreversible oxidation of regulatory proteins. The major redox changes and regulatory proteins associated with G1 and the G1/S transition are summarized in Table 2.

### 4.3. S Phase: Coordinating DNA Replication with Redox Metabolism

The S phase of the cell cycle is the most demanding phase and the requirement for ATP, reducing equivalents, and nucleotide biosynthesis peaks its maximum in this stage. Importantly, the most critical redox regulatory system of this phase is the TrxR/Trx system, which is quintessential for reduction of critical cysteine residues on RNR, the only enzyme-catalyzing synthesis of dNTPs required for DNA synthesis. Nuclear glutathione plays a vital role of maintaining the reducing environment critical for genome integrity. Critical regulators of protein-S-glutathionylation, glutaredoxins, support the process of DNA repair. Regulators of peroxide signaling, peroxiredoxins, and glutathione peroxidases perform the important function of buffering localized peroxide accumulation, ensuring physiological levels of H_2_O_2_. Thus, these redox regulatory systems act in a concerted manner to support the S phase by supplying the precursors for DNA synthesis, protecting the newly synthesized DNA against oxidative damage by maintaining the reducing environment in the nucleus, and promoting DNA repair. During S phase, the redox network shifts toward supporting DNA synthesis and genome maintenance (Table 2).

### 4.4. G2/M Transition and Mitotic Progression

Signaling pathways controlling centrosome maturation, spindle assembly, and mitotic entry need to be activated as cells are preparing for G2/M transition, and this requires increased peroxide accumulation. Interestingly, evidence has emerged that these peroxide microdomains near centrosomes and mitotic spindles are achieved through peroxiredoxins through reversible catalytic inactivation. Maintenance of localized peroxide microdomains without indiscriminate cellular increase is achieved through glutathione and glutathione peroxidases that maintain peroxide concentrations below cytotoxic thresholds without suppressing mitotic signaling. On the other hand, the literature shows that glutaredoxins are involved in the protein glutathionylation necessary for mitotic progression and cytokinesis. The mitochondrial thioredoxin system preserves mitochondrial integrity during increased respiratory activity. Together, these coordinated redox systems ensure accurate chromosome segregation while minimizing oxidative injury. A distinct redox configuration accompanies the G2/M transition and mitosis, characterized by localized peroxide signaling and increased activity of specific redox regulators (Table 2).

### 4.5. Restoration of Redox Homeostasis

Upon successful completion of cell division, glutathione redistributes from the nucleus to the cytoplasm, peroxiredoxins and glutathione peroxidases remove residual peroxide, and the thioredoxin and glutaredoxin systems restore oxidized proteins to their reduced states. This coordinated return to basal redox homeostasis terminates proliferative signaling and prepares daughter cells either for another round of division or for entry into quiescence.

### 4.6. A Unified Model of Redox-Controlled Cell-Cycle Progression

**Scheme 1:**
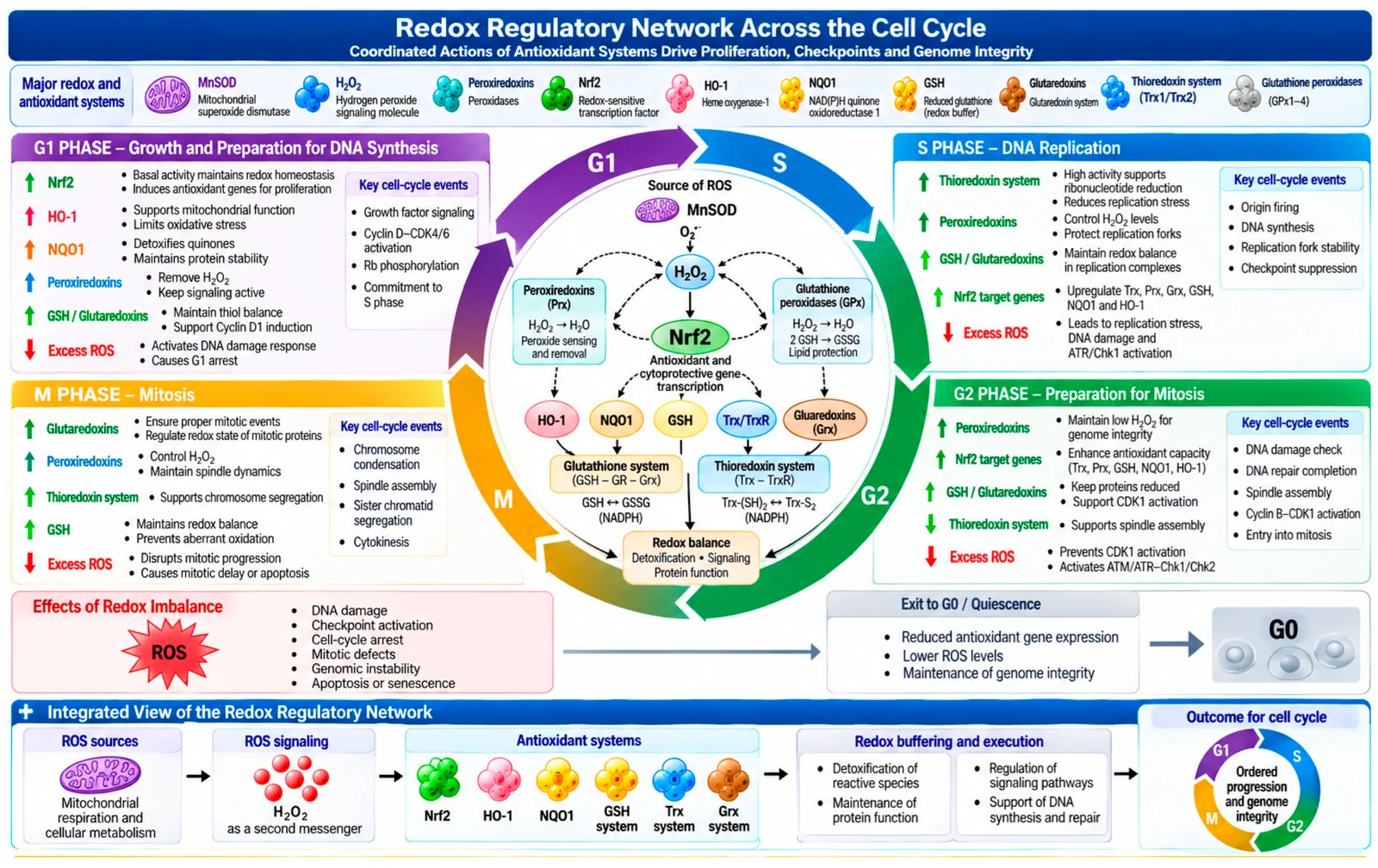
A systems-level integrated view of redox regulatory systems operating during cell-cycle progression. (Different colors distinguish cell-cycle phases and functional modules for visual clarity and do not represent quantitative differences.)

Collectively, the evidence supports a hierarchical model in which individual redox systems perform specialized yet complementary functions throughout the cell cycle. MnSOD initiates mitochondrial peroxide generation; H_2_O_2_ serves as the central signaling intermediate; peroxiredoxins regulate the spatial and temporal distribution of peroxide signals; NRF2, together with HO-1 and NQO1, coordinates transcriptional and metabolic adaptation; glutathione establishes the intracellular reducing environment; glutaredoxins regulate reversible protein S-glutathionylation; glutathione peroxidases buffer peroxide concentrations; and the thioredoxin/thioredoxin reductase system provides the reducing power required for DNA synthesis, checkpoint regulation, and completion of mitosis. The integrated redox regulatory network governing mammalian cell-cycle progression is shown in Scheme 1, and the unified redox regulatory network is shown in Figure 7. Table 2 shows cell-cycle phase-specific functions of redox regulatory systems, Table 3 lists the redox regulatory systems and their relation with specific cell-cycle phase, and Table 4 shows the context-dependent functions.

Rather than functioning independently, these systems form a highly integrated redox network in which the generation, sensing, buffering, propagation, and resolution of H_2_O_2_ signals are precisely coordinated in space and time. The collective activity of this network determines whether ROS act as physiological second messengers that promote proliferation or accumulate sufficiently to activate checkpoint pathways, senescence, or apoptosis. This systems-level perspective extends the classical Redox Code by demonstrating that redox regulatory proteins do not merely protect cells from oxidative stress but collectively orchestrate the temporal progression of the mammalian cell cycle.

## 5. Conclusions and Future Directions

The classical view of redox biology frames antioxidant systems as scavengers of ROS; however, it cannot explain the remarkable complexity of redox regulation. A careful review of the literature explains that ROS are a component of highly organized signaling cascade operated by multiple spatio-temporally segregated redox-regulatory systems. They participate in reversible oxidation–reduction reaction to control the signaling through with redox-sensitive proteins. Additionally, it becomes clear that rather than maintaining constant redox state, cycling cells constantly undergo redox fluctuations through changes in ROS generation, peroxide buffering, and thiol regulation.

Although substantial progress has been achieved, several questions remain unresolved. Much of our current understanding derives from cultured cells in which peroxide dynamics are inferred from biochemical measurements rather than directly visualized. Recent development of genetically encoded redox biosensors has begun to overcome these limitations, revealing remarkably complex peroxide oscillations within living cells. Nevertheless, whether comparable spatial organization exists in intact tissues, stem-cell niches, or regenerating organs remains incompletely understood. Likewise, the extent to which peroxide microdomains differ among physiological proliferation, tissue repair, and malignant transformation represents an important area for future investigation.

An equally important challenge for the coming decade will be to move beyond measuring ROS concentrations and toward understanding redox information flow. Future studies should focus not only on identifying which proteins undergo reversible oxidation, but also on determining how oxidative signals are encoded, transmitted between subcellular compartments, interpreted by specific protein networks, and subsequently erased. In conclusion, we propose that the mammalian cell cycle should be viewed as a redox information-processing system in which oxidative signals are continuously generated, interpreted, buffered, and resolved by an integrated network of redox regulators.

## Figures and Tables

**Table 1:** Redox regulators governing cell-cycle progression.

Redox Regulator	Cellular Localization	Major Redox Function	Major Cell-Cycle Phase	Principal Targets	Biological Outcome	Ref.
Nrf2	Cytoplasm/nucleus	Transcriptional regulator	G1/S, G2	Cyclin E, CDK2, ATR-CHK1	Redox adaptation, proliferation, checkpoints	[29,30,31]
MnSOD	Mitochondria	Superoxide → H_2_O_2_	G1, S	Cyclin D1, Cyclin E	Controls mitochondrial ROS	[16,32,33]
HO-1	Cytoplasm/ER/nucleus	Heme degradation	G1/S, G2	Cyclin D1, p21	Stress adaptation	[34,35]
NQO1	Cytoplasm	Protein stabilization	G1, G2/M	c-Fos, CKS1	G2/M progression	[36,37,38]
Peroxiredoxins	Multiple compartments	H_2_O_2_ sensing	G1, G2/M	CDK1, Cdc14B	Local peroxide signaling	[39,40]
GPXs	Cytoplasm/mitochondria	Peroxide buffering	G1/S	AMPK, ERK	Peroxide threshold	[41,42]
Glutathione	Whole cell	Redox buffer	G1/S	DNA replication	Nuclear reducing environment	[43,44,45]
Glutaredoxins	Cytoplasm/nucleus (Grx1/3); mitochondria (Grx2)	Deglutathionylation	G2/M	Protein thiols	Mitotic regulation	[46,47,48]
Thioredoxin/TrxR	Cytoplasm/mitochondria	Protein reduction	S phase	RNR, ASK1	DNA synthesis	[49,50]

**Table 2:** Cell-cycle phase-wise redox landscape.

Cell-Cycle Phase	Dominant ROS	Major Redox Proteins	Major Cell-Cycle Events	Purpose	Ref.
G1	Moderate H_2_O_2_	MnSOD, PRX, NRF2	Cyclin D induction	Cell-cycle commitment	[16,164]
G1/S	H_2_O_2_	NRF2, GSH, GPX	DNA replication begins	Prepare reducing environment	[30,165]
S	Controlled H_2_O_2_	TRX, GSH, GPX, Grx	DNA synthesis	Genome stability	[31,46,50]
G2	Rising H_2_O_2_	NRF2, PRX	DNA repair	Checkpoint activation	[39,166]
M	Local H_2_O_2_	PRXI, GRX3, TRX2	Spindle assembly	Chromosome segregation	[27,58,151]
Cytokinesis	Declining ROS	GPX, GSH, TRX	Return to basal state	Homeostasis	[24,167]

**Table 3:** Redox proteins and their cell-cycle targets.

Redox Protein	Cyclin/CDK Target	DNA Repair Target	Signaling Target	Outcome	Ref
NRF2	Cyclin E/CDK2	RAD51	ATR (proposed target)	G1/S, DNA repair	[29,31]
HO-1	Cyclin D1	—	VEGF	Proliferation	[34,168]
NQO1	Cyclin D1, Cyclin B1	P53	c-FOS	G2/M	[36,137]
MnSOD	Cyclin D1, Cyclin E	—	NFκB	Quiescence	[16,66]
TRX	—	RNR	ASK1	DNA synthesis	[50,160]
PRX	CDK1	CDC14B	PTPs	Mitotic control	[15,39,166]
GPX	Cyclin D	—	AMPK	Redox buffering	[41,42,147]
GRX	—	RNR	Protein glutathionylation	Mitosis	[48,49,151,152]

**Table 4:** Context-dependent functions of redox proteins.

Protein	Physiological Proliferation	Oxidative Stress	Cancer	Ref.
NRF2	Promotes G1/S	Arrest	Tumor survival	[29]
HO-1	Cell survival	Repair	Therapy resistance	[169,170,171,172]
NQO1	Proliferation	p53 stabilization	Tumor growth	[36,138]
MnSOD	Controlled proliferation	Quiescence	Tumor suppressor	[65,173]
GPX	DNA protection	Prevents ROS	Ferroptosis resistance	[41,174]
TRX	DNA synthesis	Repair	Drug resistance	[50,175]
GRX	Protein repair	Checkpoints	Tumor progression	[46,47]

## Data Availability

No new data were created or analyzed in this study. Data sharing is not applicable to this article.

## Abbreviations

AI, artificial intelligence; AMPK, AMP-activated protein kinase; APC/C, anaphase-promoting complex/cyclosome; ARE, antioxidant response element; ASK1, apoptosis signal-regulating kinase 1; ATR, ataxia telangiectasia and Rad3-related protein; CDK, cyclin-dependent kinase; CHK1, checkpoint kinase 1; CKS1, CDC28 protein kinase regulatory subunit 1; EGF, epidermal growth factor; ER, endoplasmic reticulum; ERK, extracellular signal-regulated kinase; FOXM1, forkhead box M1; GCLC, glutamate-cysteine ligase catalytic subunit; GCLM, glutamate-cysteine ligase modifier subunit; GPX, glutathione peroxidase; GR, glutathione reductase; Grx, glutaredoxin; GSH, reduced glutathione; GSSG, oxidized glutathione; H_2_O_2_, hydrogen peroxide; HDGF, hepatoma-derived growth factor; HIF-1α, hypoxia-inducible factor 1-alpha; HO-1, heme oxygenase-1; JNK, c-Jun N-terminal kinase; MAPK, mitogen-activated protein kinase; MnSOD, manganese superoxide dismutase; NAD^+^, oxidized nicotinamide adenine dinucleotide; NADPH, reduced nicotinamide adenine dinucleotide phosphate; NF-κB, nuclear factor kappa B; NQO1, NAD(P)H:quinone oxidoreductase 1; NRF2, nuclear factor erythroid 2-related factor 2; PCNA, proliferating cell nuclear antigen; PERK, protein kinase R-like endoplasmic reticulum kinase; PI3K, phosphoinositide 3-kinase; PP2A, protein phosphatase 2A; PRX, peroxiredoxin; PTP1B, protein tyrosine phosphatase 1B; RAD51, RAD51 recombinase; RNR, ribonucleotide reductase; ROS, reactive oxygen species; SIRT2, sirtuin 2; SOD2, superoxide dismutase 2; Trx, thioredoxin; TrxR, thioredoxin reductase; VEGF, vascular endothelial growth factor.
